# Cost Implications of Comorbidity for Autologous Stem Cell Transplantation in Elderly Patients with Multiple Myeloma Using SEER-Medicare

**DOI:** 10.1155/2016/3645623

**Published:** 2016-10-18

**Authors:** Gunjan L. Shah, Aaron Winn, Pei-Jung Lin, Andreas Klein, Kellie A. Sprague, Hedy P. Smith, Rachel Buchsbaum, Joshua T. Cohen, Kenneth B. Miller, Raymond Comenzo, Susan K. Parsons

**Affiliations:** ^1^Adult Bone Marrow Transplantation Service, Department of Medicine, Memorial Sloan-Kettering Cancer Center, 1275 York Box 298 Avenue, New York, NY 10065, USA; ^2^Center for the Evaluation of Value and Risk in Health, Institute for Clinical Research and Health Policy Studies, Tufts Medical Center, 800 Washington St., Box 63, Boston, MA 02111, USA; ^3^Division of Hematology/Oncology, Department of Medicine, Tufts Medical Center, 800 Washington St., Box 245, Boston, MA 02111, USA; ^4^Center for Health Solutions, Institute for Clinical Research and Health Policy Studies, Tufts Medical Center, 800 Washington St., Box 345, Boston, MA 02111, USA

## Abstract

Comorbidity is more common in older patients and can increase the cost of care by increasing toxicity. Using the SEER-Medicare database from 2000 to 2007, we examined the costs and life-year benefit of Auto-HSCT for MM patients over the age of 65 by evaluating the difference over time relative to comorbidity burden. One hundred ten patients had an Auto-HSCT in the early time period (2000–2003) and 160 in the late time period (2004–2007). Patients were divided by a Charlson Comorbidity Index (CCI) of 0 or greater than 1 (CCI1+). Median overall survival was 53.5 months for the late time period patients compared to 40.3 months for the early time period patients (*p* = 0.031). Median costs for CCI0 versus CCI1+ in the early period were, respectively, $70,900 versus $72,000 (100 d); $86,100 versus $98,300 (1 yr); and $139,200 versus $195,300 (3 yrs). Median costs for late period were, respectively, $58,400 versus $60,400 (100 d); $86,300 versus $77,700 (1 yr); and $124,400 versus $110,900 (3 yrs). Comorbidity had a significant impact on survival and cost among early time period patients but not among late time period patients. Therefore, older patients with some comorbidities can be considered for Auto-HSCT depending on clinical circumstances.

## 1. Introduction

Medicare coverage of Auto-HSCT for MM began in 2000. By 2009, patients over the age of 60 accounted for 40% of Auto-HSCT procedures, with the leading indication being MM [1]. Older cancer patients are known to have more preexisting health conditions [2], and higher comorbidity assessment before transplant in MM is correlated with increased toxicity [3], worse survival [4], and longer length of stay [5]. The cost implications of the rise in Auto-HSCT for the elderly are not well described, with most studies focusing on younger patients at single institutions [6–10]. Using the Surveillance, Epidemiology, and End Results- (SEER-) Medicare database from 2000 to 2007, we examined the costs and life-year benefit of Auto-HSCT for MM patients over the age of 65 by evaluating the difference over time relative to comorbidity burden.

## 2. Patients and Methods

### 2.1. Data Source

SEER registries include the Alaska Native Tumor Registry, Arizona Indians, Cherokee Nation, Connecticut, Detroit, Georgia (Atlanta, Greater Georgia, and Rural Georgia), California (Greater Bay Area Cancer Registry, Los Angeles, and Greater California), Hawaii, Iowa, Kentucky, Louisiana, New Jersey, New Mexico, Seattle-Puget Sound, and Utah. For each cancer diagnosis collected by a SEER registry, demographics and selected diagnostic and treatment information, along with date and cause of death, are collected. Currently, the SEER registries cover approximately 28% of the United States population and have an internal quality control process to ensure the accuracy of the data [11]. Among the patients who are at least 65 years old in the SEER registry, 93% have been linked to their fee-for-service (FFS) Medicare claims [12, 13]. The linkage completed in 2011 includes SEER incident cases through December 31, 2007, and Medicare claims through December 31, 2009. The Patient Entitlement and Diagnosis Summary (Medicare enrollment information), the Medicare Provider Analysis and Review (MEDPAR) (inpatient Medicare Part A claims), the National Claims History (NCH) file (provider Medicare Part B claims), and the Outpatient (institutional Medicare Part B claims) files, Durable Medical Equipment files, Hospice files, and Home Health files were used in this analysis. The study was approved by the Tufts Medical Center Institutional Review Board.

### 2.2. Patient Sample

We identified patients with MM in SEER by the International Classification of Disease-Oncology Version 3 (ICD-O-3) code, 9731-2, restricting our study to cases diagnosed after October 2000, when Medicare coverage of Auto-HSCT began. We used International Classification of Disease-9 (ICD-9, 41.00, 41.01, 41.04, 41.07, 41.09) or Healthcare Common Procedure Coding System (HCPCS, 38241) codes to identify patients as having an Auto-HSCT after MM diagnosis. We included only those cases enrolled in Medicare Part A and B FFS plans and limited attention to individuals over age 65 at diagnosis in order to calculate the comorbidity index. We then divided cases into an early time period (diagnosed October 1, 2000–December 31, 2003) and late time period (diagnosed January 1, 2004–December 31, 2007). We chose these dates to facilitate comparison of outcomes for patients before and after the introduction of bortezomib, which we hypothesized would add substantial cost compared to the previous therapeutic option of vincristine, doxorubicin, and dexamethasone. Bortezomib was FDA-approved in June 2003, with Center for Medicare Services (CMS) pass-through billing starting January 1, 2004 [14].

### 2.3. Comorbidity

We modified calculation of the original Charlson Comorbidity Index (CCI) using ICD-9 codes from Medicare claims in the year prior to MM diagnosis [15]. We divided cases into two groups: those individuals with a CCI of 0 (no comorbidities) and those with a CCI score of 1 or more (designated “1+”). This cutoff was chosen due to the small number of patients with more than one comorbidity (Table 1). In addition, as comorbidity can be underrepresented by billing data [15, 16] any comorbidity was considered significant.

### 2.4. Statistical Methods

All statistical analyses were performed using SAS (version 9.3, SAS, Cary, NC), Stata (version 12, StataCorp, College Station, TX), and Excel (Microsoft, Redmond, WA). Demographic characteristics were compared using two-sided *t*-tests and chi-squared tests.

We determined the date of death from the SEER Patient Entitlement and Diagnosis Summary File. We calculated survival for the first 100 days (d), 1 year (yr), and 3 yrs after diagnosis using Kaplan-Meier curves. We included up to 9 years of follow-up data and calculated the log rank test for comparison. Using the medical care component of the Consumer Price Index, we inflation-adjusted cost data, converting it to 2010 US dollars [17, 18]. We then calculated costs for the first 100 days (d), 1 year (yr), 3 yrs, and 5 yrs after diagnosis along with the cumulative cost since diagnosis. Cumulative per patient costs were calculated, averaged, and plotted to compare the total costs in the two time periods.

## 3. Results

### 3.1. Cohort Characteristics

Of the 22,286 patients in SEER-Medicare who were diagnosed with MM between 2000 and 2007, 6,078 (27%) were over the age of 65 and enrolled in Medicare Part A and B FFS. Of these, 270 (4.4%) met our billing code criteria for having undergone an Auto-HSCT (110, early; 160, late). Characteristics between time periods did not differ statistically (Table 1). Forty percent of the subjects in each time period were female and most were Caucasian (88%, early; 91%, late). The median age in both time periods was 70 years (range: 66–92 yrs (early); range: 66–90 yrs (late)). Only a small portion of patients was over 81 yrs (3% versus 1% for the early and late time periods, resp.). Comorbidity did not differ over time. The CCI was 0 for 62% early time period subjects and 63% for late time period subjects.

### 3.2. Survival

**Figure 1:**
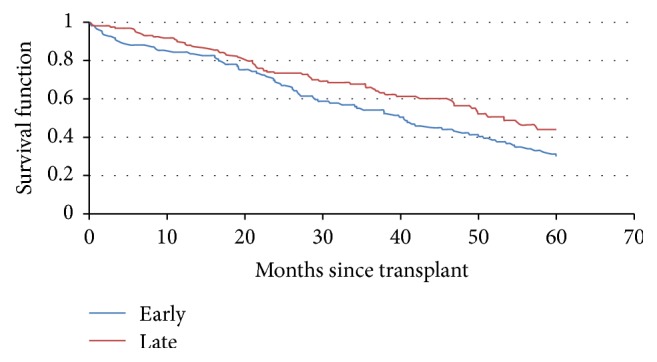
Overall survival after transplant. Early transplants are those from 2000 to 2003, while late transplants are between 2004 and 2007. Median overall survival for late time period patients was 53.3 months and for early time period patients was 40.3 months (*p* = 0.031).

The median time to transplant was significantly longer for late time period subjects (277 d) than for early period subjects (223 d) (*p* = 0.03). More than 96% of transplants occurred within 3 years of diagnosis for patients in both time periods. While survival after Auto-HSCT in the early time was associated with comorbidity level, this pattern was not observed in the late time period (Table 1). Median overall survival was significantly longer for late time period patients (53.3 months) than for early time period patients (40.3 months) (*p* = 0.031, Figure 1). The subset of patients over the age of 70 had a 5-year OS of 25% in the early time period compared to 65% for the late group.

### 3.3. Cost

**Figure 2:**
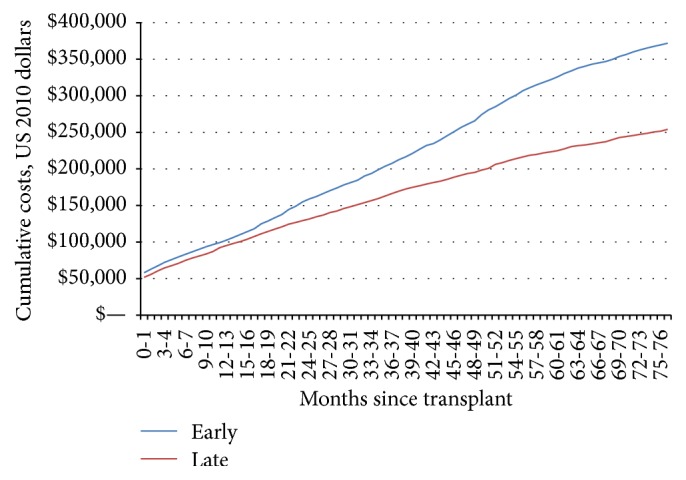
Average cumulative per person cost. Early transplants are those from 2000 to 2003, while late transplants are between 2004 and 2007. The cumulative cost was lower for patients transplant in the later time period compared to the earlier.

As with survival, higher comorbidity scores were associated with an increased cost among early period patients, whereas, for late period patients, costs did not differ statistically between CCI0 and CCI1+ patients (Table 1). Median costs for CCI0 versus CCI1+ in the early period were, respectively, $70,900 versus $72,000 (100 d); $86,100 versus $98,300 (1 yr); and $139,200 versus $195,300 (3 yrs). Median costs for the late period were, respectively, $58,400 versus $60,400 (100 d); $86,300 versus $77,700 (1 yr); and $124,400 versus $110,900 (3 yrs). In addition, the average per person cumulative cost remained lower for late period patients for at least six years after transplant (Figure 2). Finally, using quantile regression, stratifying by age (>70 versus ≤70) and comorbidity did not change the cost in either the early or late time period.

## 4. Discussion

Although recent advances in the management of MM have enhanced responses [19, 20] and extended survival [21, 22] the cost of these advancements has not been evaluated in elderly patients. As contemporary therapy has allowed a greater percentage of older patients to become transplant eligible, the impact of comorbidities upon survival must be taken into account. In a recent study by Saad et al., the application of the hematopoietic cell transplant comorbidity index (HCT-CI) to Auto-HSCT revealed that higher HCT-CI correlated with inferior survival, but not with nonrelapse mortality [4]. Studies have reported both positive [23, 24] and negative [25–27] correlations between cost and comorbidity in patients with solid tumors, but similar studies have not been conducted in patients with hematologic malignancies.

This is the first paper to compare the costs of Auto-HSCT and comorbidity in elderly patients with MM in the era of novel agents, notably bortezomib. Our findings showed that comorbidities were significantly associated with higher health care costs in the early time period, but not afterwards. The lack of association between cost and comorbidity in the late period may be the result of improved responses with less pre-Auto-HSCT therapy. In particular, patients undergoing Auto-HSCT in the later time period may have experienced lower rates of treatment-induced toxicity secondary to comorbidities.

Our results, demonstrating lower 100-day survival rates than previously published [28, 29], may be due to the exclusion of high volume centers in SEER or the inclusion of older patients. However, Majhail et al. found, using Thomson Reuters MarketScan data, that 8% of MM and non-Hodgkin's lymphoma patients over age 60 disenrolled from private insurance within 100 days of Auto-HSCT, which they considered a surrogate for mortality [30]. The Majhail et al. results are comparable to those with CCI1+ in the later time period. One-year survival in the more recent time frame for those with no comorbidities is also consistent with the recently published Center for International Blood and Marrow Transplant Research (CIBMTR) analysis [31]. Three-year survival rates in the late period are also comparable to survival reported by the CIBMTR [1] and a meta-analysis of three international trials [32]. The improvement is likely related to better supportive care [33] and improved response rates with novel agents [22].

First 100-day costs in our study ranged from medians of $58,400 to $78,000 depending on time period and comorbidity. In a study looking at a national private claims database, median 100-day costs were $90,000 for MM patients [30]. Furthermore, they found that median costs were lower for ages 41–60 yrs than for ages 21–40 yrs, which mirror our results. Comparison to other studies with younger patients is difficult given that most occur outside the US, did not include novel agents, and did not evaluate comorbidity [6, 10, 34, 35]. However, the costs in our study are similar to those found in a study of the Nationwide Inpatient Sample where the mean age was 55 years [36].

We acknowledge our study's limitations. First, because Medicare Part D prescription coverage for oral prescriptions did not begin until 2009, costs for oral medication (primarily lenalidomide and thalidomide, FDA-approved in 2006) were not included in our analysis. Future studies will be needed to address the contribution of oral therapies to the overall cost of care. In contrast, costs associated with bortezomib use are included. Second, claims databases often underrepresent comorbidity [15], although this issue may not have biased our analysis since the information would presumably be missing from both groups we compared (patients from the early and late time periods). Finally, because SEER does not collect the laboratory values, disease burden, or risk classification for MM, we cannot compare treatment response or disease-free survival between the two time periods. Inclusions of Durie Salmon staging was initiated only in 2011 and the International Staging System is still not captured in SEER [11, 37–40]. Further evaluation of clinical trial or registry-based data may help to provide specific circumstances in which Auto-HSCT may be more or less cost effective, based on prognostic factors or response to initial therapy.

Despite these limitations, the strength of this analysis is the use of a nationally representative sample of patients, allowing for generalizability of our findings [41]. The link of SEER registry data (i.e., diagnosis, date of diagnosis, and updated survival information) with comprehensive cost date eliminates many of the shortcomings of other administrative databases, such as loss of coverage due to insurance churning, or the use of single institutional experience. The use of Medicare data allows capture of longitudinal claims from diagnosis until death in any state or institution where the interventions took place.

## 5. Conclusion

Overall, we found that the median time to transplant was significantly longer in late time period patients. Comorbidity was significantly associated with worse survival and higher health care costs in early time period patients, but not the late time period patients. Finally, with a similar comorbidity distribution over time, late time period patients had significantly extended survival and lower cumulative costs, compared to early time period patients.

## Figures and Tables

**Table 1:** Patient characteristics. Early transplants are those from 2000 to 2003, while late transplants are between 2004 and 2007.

	Early		Late	
*Patient characteristics*	*N* = 110, (%)		*N* = 160, (%)	
Female	45 (41)		64 (40)	
White	97 (88)		145 (91)	
Age, median (range)	70 (66–92)		70 (66–90)	
66–70	75 (68)		100 (63)	
70–74	21 (19)		47 (29)	
75+	14 (13)		13 (8)	
Charlson Comorbidity Index				
0	68 (62)		101 (63)	
1	29 (26)		37 (23)	
2+	13 (12)		22 (14)	

Survival outcomes	CCI0	CCI1+	CCI0	CCI1+
100 days^*∗*^	95%	85%	99%	93%
1 year^*∗∗*^	90%	76%	92%	86%
3 years^*∗∗∗*^	62%	42%	66%	66%

Cost outcomes Median (25%; 75%)	CCI0	CCI1+	CCI0	CCI1+
100 days	*N* = 65 $70,900 (46,000; 85,200)	*N* = 36 $72,000 (55,600; 81,100)	*N* = 100 $58,400 (37,900; 73,900)	*N* = 54 $60,400 (50,300; 70,100)
1 year	*N* = 61 $86,100 (59,500; 113,100)	*N* = 31 $98,300 (75,100; 116,700)	*N* = 93 $81,300 (59,400; 112,300)	*N* = 49 $77,700 (69,300; 91,800)
3 years	*N* = 42 $139,200 (94,200; 179,900)	*N* = 17 $195,300 (153,300; 224,600)	*N* = 45 $124,400 (97,300; 159,500)	*N* = 29 $110,900 (89,600; 167,300)

^*∗*^Early *p* = 0.06, late *p* = 0.098. ^*∗∗*^Early *p* = 0.043, late *p* = 0.20. ^*∗∗∗*^Early *p* = 0.34, late *p* = 0.86.
